# A Rare Case of Hypersensitivity Pneumonitis due to Florida Red Tide

**DOI:** 10.1155/2019/1934695

**Published:** 2019-07-14

**Authors:** Raju Reddy, Nupur Verma, Tan-Lucien Mohammed

**Affiliations:** ^1^Division of Pulmonary, Critical Care and Sleep Medicine, Department of Internal Medicine, University of Florida, 1600 SW Archer Rd, P.O. Box 100225, Gainesville, FL, USA; ^2^Department of Radiology, University of Florida, 1600 SW Archer Rd, P.O. Box 100374, Gainesville, FL, USA

## Abstract

Florida red tides occur annually due to proliferation of the marine dinoflagellate* Karenia brevis*, which produces neurotoxins known as brevotoxins. Inhalational exposure to brevotoxins usually results in upper airway symptoms only. Rarely does exposure lead to lower respiratory tract symptoms as in our case. We report a case of a 50-year-old man who presented with a 4-week history of dyspnea after exposure to the red tide. Computed tomography (CT) of the chest showed diffuse bilateral ground glass opacities and interstitial thickening. Bronchoalveolar lavage cultures and cytology were negative. The patient was started on steroids. Over the next few weeks, the patient's symptoms resolved. Repeat CT chest showed complete resolution of the ground glass opacities. Steroids were then tapered. Most patients who are exposed to algal blooms have self-limiting symptoms. Patients with asthma are particularly susceptible to worsening respiratory symptoms after exposure to brevotoxin aerosols. This case highlights that, in rare cases, exposure to red tide can results in severe lower respiratory tract symptoms.

## 1. Case Presentation

**Figure 1:**
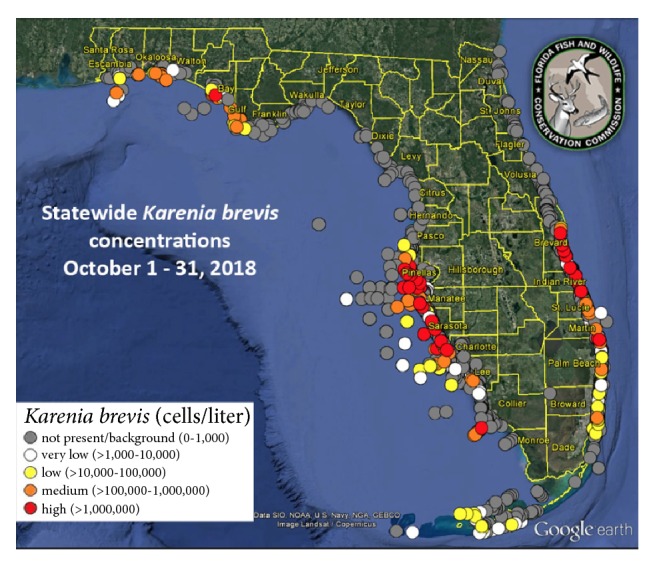
Concentrations of* Karenia brevis* during exposure. Image source: Florida Fish and Wildlife Conservation Commission.

**Figure 2:**
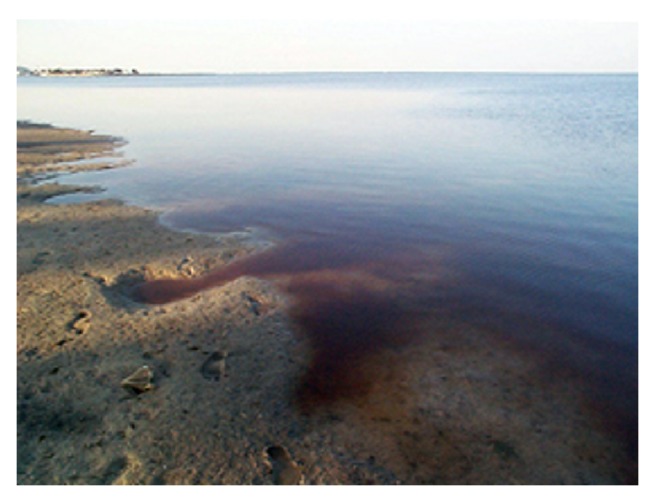
*Karenia brevis* proliferation. Source: Florida Fish and Wildlife Commission.

A 50-year-old man with a history of hypertension, 30-pack year tobacco history and pulmonary embolism 12 years prior to admission presented with a 4-week history of progressively worsening dyspnea. His symptoms began with a dry cough, mild dyspnea and fevers. He was given a course of levofloxacin and azithromycin. While his cough and fevers resolved, his dyspnea continued to worsen. These symptoms began after he was exposed to the Florida red tide. Four weeks earlier, the patient reported going to the beach for a swim. Unfortunately, he swam into a large area of red tide (Figure 1). Florida red tides occur due to high concentrations of algal blooms, particularly* Karenia brevis (K. brevis)* which gives the water a red discoloration (Figure 2). Immediately after the exposure, the patient began to have symptoms. He denied any recent travel, illicit drug use and occupational exposures. He also denied receiving any blood transfusions recently. He worked as a nurse.

On initial examination, the patient was profoundly hypoxic requiring FiO2 0.8 to maintain an appropriate oxygen saturation via high flow nasal canula. He was ill appearing and was in mild respiratory distress. The remainder of the examination was significant for bibasilar crackles and absence of pedal edema. Initial labs were significant for white blood cell count at 28 x 10^3^ cells/*μ*L with neutrophilia and absence of eosinophilia. Other significant labs included elevated brain natriuretic peptide 440 pg/mL (normal < 100 pg/mL) and normal procalcitonin 0.22 ng/mL (normal < 0.5 ng/mL) levels. The patient was started on broad spectrum antibiotics.

**Figure 3:**
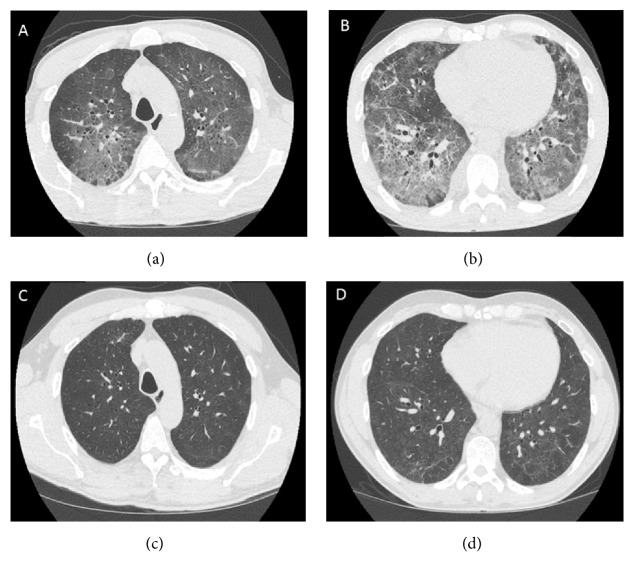
(a) and (b) At the time of presentation, axial computed tomography of the chest images show upper lobe predominant ground glass attenuation, mild interlobular septal thickening and small cysts. (c) and (d) Eight weeks following treatment, axial computed tomography of the chest images shows near complete resolution of ground-glass attenuation, interlobular septal thickening and cysts.

Computed tomography (CT) of the chest showed a lower predominant ground-glass attenuation, along with small cysts and smooth interlobular septal thickening (Figures 3(a) and 3(b)). There were no centrilobular nodules. A bronchoscopy was performed on the day of admission. The patient was not a candidate for a transbronchial biopsy due to high oxygen requirements. Bronchoalveolar lavage (BAL) fluid analysis showed 88% macrocytes and 10% lymphocytes. The patient was continued on anti-bacterial therapy while further work-up continued. Despite a weeklong course of board spectrum antibiotics, he continued to have high oxygen requirements. At this point, differential diagnosis included acute hypersensitivity pneumonitis (HP) due to red-tide exposure, respiratory bronchiolitis associated interstitial lung disease (RB-ILD), desquamative interstitial pneumonia (DIP) and acute respiratory distress syndrome (ARDS). The remainder of the BAL fluid analysis was negative for bacterial and fungal cultures, viral studies including influenza and cytology noted no organisms or malignant cells. Other negative findings included an autoimmune work-up for connective tissue disease including an extended myositis panel and hypersensitivity panel.

Since the cultures were negative, the patient was started on Prednisone 1 mg/kg daily with a diagnosis of HP due to red tide exposure. While RB-ILD and DIP remained in consideration, the patient had a clear temporal association of symptom onset after an inhalational exposure. Furthermore, the patient had not smoked for the last 4 weeks prior to presentation at our institution. Despite being off cigarettes, his dyspnea continued to worsen. Of note, BAL findings in patients with RB-ILD or DIP are typically macrocyte predominant similar to our patient, whereas in HP, BAL findings are lymphocytic predominant. However, smokers with HP may not have a marked BAL lymphocytosis compared to non-smokers [1]. In addition, up to two-thirds of patients with RB-ILD or DIP improve once they quit smoking [2]. In cases where they do not improve with quitting smoking, corticosteroids provide only marginal benefit [3]. Our patient however noted a dramatic improvement with steroids. Together, the initial symptom onset after exposure, persistence of symptoms despite quitting smoking and improvement with steroids, suggested that the patient developed an acute case of HP following exposure to the red tide. CT chest obtained 8 weeks after initial presentation showed significant improvement of the ground glass attenuation and interstitial thickening (Figures 3(c) and 3(d)). The patient was gradually weaned off steroids over the next 4 weeks. While most cases of red tide exposure lead to upper airway symptoms only, this case highlights that patients may also develop lower airway symptoms.

## 2. Discussion

Florida red tides occur due to proliferation of the toxic dinoflagellate,* K*.* brevis*.* K*.* brevis* is a unicellular organism found widely in the Gulf of Mexico [4]. It produces PbTx-2 and PbTx-3, the major brevotoxins that are responsible for both neurologic symptoms if ingested and upper airway symptoms if inhaled [5, 6]. The tide peaks in late summer to early fall and can last from days to months. The longest documented red tide bloom lasted for 30 months from 1994 to 1997 [7]. The current red tide bloom began in October 2017, peaked from July 2018 to October 2018 and ended in March 2019 [8]. Our patient was exposed to red tide during a period of high concentration (Figure 1). While the annual number of cases of brevotoxin exposure is unknown, studies show an increase in emergency room visits for respiratory symptoms during peak periods [9]. Kirkpatrick* et al.* found a 19% increase in rates of pneumonia diagnoses during a red tide period compared to a non-red tide period.

Typically, exposure to brevotoxins results in upper airway symptoms only. This is because average size of the aerosolized toxin is 8-9*μ* whereas particles need be less than 5*μ* to enter the lower respiratory tract [10]. In one study of 27 healthy individuals exposed to low level brevotoxins, 35% developed throat irritation, 53% developed cough, and 23% developed nasal congestion whereas on days with higher levels of brevotoxins, 54% developed throat irritation, 77% developed cough and 23% developed nasal congestion [11]. In addition, study participants had a slight reduction in peak expiratory flows and forced expiratory volume-1 (FEV1) on exposed days compared to unexposed days but no reduction in their exercise capacity. Other studies have found a direct correlation between symptoms and exposure burden with higher levels of toxin in the air leading to lower respiratory tract involvement [12]. Our patient likely developed lower respiratory tract symptoms because he had a heavy exposure burden. He was exposed to not only the aerosolized toxin on the beach but also brevotoxins in the water while swimming.* K. brevis* is easily lysed and thus releases toxin in to the water. Of note, the brevotoxin is stored in clams and oysters which accounts for the neurotoxic poisoning in people who consume contaminated shellfish.

Of particular concern is exposure of brevotoxin in uncontrolled asthmatics. In a study of 97 patients with self-reported asthma, there was significant reduction in FEV1 after only 1 hour of exposure to the aerosolized toxin [13]. These findings were particularly more prominent in patients who used their inhalers more frequently. In another study of 125 asthmatics, 38 patients were more reactive to the toxin. These patients had larger reductions in FEV1 following exposure [14]. In addition, prior to enrollment, these patients were using asthma medications more frequently and were more likely to have been hospitalized for respiratory symptoms in the last 12 months. Together, these findings suggest that patients who have uncontrolled asthma may be more susceptible to developing respiratory symptoms following exposure to the brevotoxin.

Exposure to the inhaled toxin results in mass cell degranulation and histamine release resulting in elevated levels of inflammatory cytokines such as interleukin-6 which may account for the respiratory symptoms [12]. From a public health standpoint, preventing exposure to the toxin is critical. Humans can be exposed through food, water and air [15]. The brevotoxin is odorless, tasteless and heat stable [15]. During periods of algal bloom, the public and particularly asthmatics must be counseled to stay indoors since the aerosolized toxin can travel up to 2 miles inland depending on the wind conditions [16]. Therefore, asthmatic patients living inland should remain aware of potential exposure in the right conditions. Other measures to minimize exposure include wearing a paper surgical mask to decrease exposure [5, 16].

This case highlights the respiratory pathology associated with red tide exposure. The majority of cases are limited to upper respiratory tract infection. However, with a large exposure burden, the lower respiratory tract can be involved as was our case.
